# A Deep Learning Perspective on Dropwise Condensation

**DOI:** 10.1002/advs.202101794

**Published:** 2021-09-24

**Authors:** Youngjoon Suh, Jonggyu Lee, Peter Simadiris, Xiao Yan, Soumyadip Sett, Longnan Li, Kazi Fazle Rabbi, Nenad Miljkovic, Yoonjin Won

**Affiliations:** ^1^ Department of Mechanical and Aerospace Engineering University of California, Irvine 5200 Engineering Hall Irvine CA 92617–2700 USA; ^2^ Department of Mechanical Science and Engineering University of Illinois at Urbana‐Champaign Urbana IL 61801 USA; ^3^ Department of Electrical and Computer Engineering University of Illinois at Urbana‐Champaign Urbana IL 61801 USA; ^4^ Materials Research Laboratory University of Illinois at Urbana‐Champaign Urbana IL 61801 USA; ^5^ International Institute for Carbon Neutral Energy Research (WPI‐12CNER) Kyushu University 744 Moto‐oka, Nishi‐ku Fukuoka 819‐0395 Japan

**Keywords:** AI computer vision, deep learning, droplet statistics, dropwise condensation, real‐time heat transfer mapping

## Abstract

Condensation is ubiquitous in nature and industry. Heterogeneous condensation on surfaces is typified by the continuous cycle of droplet nucleation, growth, and departure. Central to the mechanistic understanding of the thermofluidic processes governing condensation is the rapid and high‐fidelity extraction of interpretable physical descriptors from the highly transient droplet population. However, extracting quantifiable measures out of dynamic objects with conventional imaging technologies poses a challenge to researchers. Here, an intelligent vision‐based framework is demonstrated that unites classical thermofluidic imaging techniques with deep learning to fundamentally address this challenge. The deep learning framework can autonomously harness physical descriptors and quantify thermal performance at extreme spatio‐temporal resolutions of 300 nm and 200 ms, respectively. The data‐centric analysis conclusively shows that contrary to classical understanding, the overall condensation performance is governed by a key tradeoff between heat transfer rate per individual droplet and droplet population density. The vision‐based approach presents a powerful tool for the study of not only phase‐change processes but also any nucleation‐based process within and beyond the thermal science community through the harnessing of big data.

## Graphical abstract

A vision‐based framework utilizing artificial intelligence is proposed to meet the challenges in acquiring physical descriptors of dropwise condensation. Using this framework, the study investigates the relationship between droplet statistics and heat and mass transfer with unprecedented spatio‐temporal resolutions. The results show the importance of codesigning heat transfer rate per droplet and droplet number density to optimize heat transfer performance.

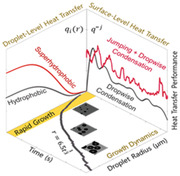

## 1 Introduction

Condensation is an efficient mass‐transfer process observed in nature and is essential to many industrial applications such as thermoelectric and nuclear power generation,^[^ ^1^ ^]^ water harvesting systems,^[^ ^2^, ^3^, ^4^, ^5^, ^6^ ^]^ heat exchangers,^[^ ^7^, ^8^ ^]^ and desalination plants.^[^ ^9^, ^10^ ^]^ Condensation involves the nucleation of droplets on a surface that can lead to significant heat and mass transfer improvement depending on the droplet dynamics.^[^ ^11^ ^]^ Highly efficient dropwise condensation on nonwetting surfaces is characterized by the formation of discrete droplets that nucleate, grow, and depart from the surface in a cyclic manner.^[^ ^12^ ^]^ While traditional dropwise condensing surfaces rely on gravity to remove droplets having high Bond number (Bo ≈ 1), recent advances in surface structuring and nanomanufacturing have enabled droplet removal through unique condensate behavior such as coalescence‐induced droplet jumping at Bo ≈ 0.001,^[^ ^11^, ^12^, ^13^, ^14^, ^15^, ^16^, ^17^ ^]^ and the synergistic combination of droplet and film dynamics.^[^ ^18^ ^]^

The dynamic behavior of individual droplet–droplet and droplet–surface interactions are the main contributing parameters that determine the local and overall heat and mass transfer.^[^ ^16^ ^]^ Despite efforts to quantify condensation performance by linking droplet statistics with classical phase change theories,^[^ ^19^ ^]^ obtaining the requisite data needed at small scales (≈ µm) remains a challenge.^[^ ^20^, ^21^, ^22^, ^23^, ^24^, ^25^ ^]^ Using physical sensors is impractical for collecting information due to the need for thousands of images, with each image containing thousands of droplets, totaling more than a million individual interactions for a one‐hour experiment. As a result, the alternative approach for instance‐level quantification has been leveraging standard computer vision algorithms such as thresholding, *k*‐means clustering, edge detection and Voronoi diagrams.^[^ ^26^, ^27^ ^]^ These traditional computer vision methods offer swift analysis of multiple objects, but are unsuitable for the analysis of long‐duration condensation experiments because they require strict environmental control and human intervention to extract reliable features from image datasets.^[^ ^28^ ^]^

The development of precise and automatic object prototyping of instances that can provide physical descriptors would represent a game‐changing innovation for thermofluidic engineering. A promising avenue to address these challenges is to use emerging artificial intelligence (AI)‐based computer vision techniques to track and analyze moving objects. Most AI‐based computer vision systems primarily leverage deep convolutional neural networks (DCNNs), which emulate the human visual cortex by learning features through multiple operational layers.^[^ ^29^ ^]^ The number of operational layers renders the DCNN depth, and the layer structure constitutes the DCNN architecture.^[^ ^30^ ^]^ DCNNs provide the benefit of being spatially invariant, meaning that image features can be recognized without being sensitive to their position in the image. This advantage helps DCNNs easily generalize to new imaging conditions and allows them to significantly outperform conventional image analysis methods.^[^ ^31^ ^]^ Past work has shown the promise of DCNNs in thermofluidic sciences by demonstrating an autonomous framework that predicts heat transfer performance using hierarchical image features.^[^ ^32^ ^]^ Among other widespread use of DCNNs to tackle visual tasks,^[^ ^33^, ^34^, ^35^, ^36^, ^37^ ^]^ object tracking; the task of following objects through a series of time‐lapse images has proven to be useful for a variety of different fields including plant phenotyping,^[^ ^38^ ^]^ surface engineering,^[^ ^39^ ^]^ drug delivery,^[^ ^40^ ^]^ and wildlife monitoring.^[^ ^41^, ^42^ ^]^ Object tracking primarily operates based on two subtasks: object detection and object linkage. Depending on the dataset, different object detection modules can be chosen, varying from bounding box detection,^[^ ^43^ ^]^ semantic segmentation,^[^ ^44^ ^]^ and instance segmentation.^[^ ^45^ ^]^ Linking algorithms enables the acquisition of spatio‐temporal features by connecting principal features of the detected objects.^[^ ^46^ ^]^

Herein, we present a powerful metrology tool to systematically study condensation by rationally converging thermofluidic imaging techniques, intelligent vision, and deep learning. The vision‐based framework enables the autonomous curation of >100 000 physical descriptors per dataset for holistic analysis of condensation phenomena and heat flux mapping with extreme spatial (≈300 nm) and temporal (≈200 ms) resolutions. We use the deep learning framework to unveil mechanistic understanding of the relation between droplet statistics, local heat and mass transfer, and overall heat and mass transfer performance. Our experimental findings suggest design rules for next‐generation condensing surfaces and coatings by establishing the trade‐off between heat transfer rate per individual droplet and droplet population density through data‐driven analysis. Our deep learning strategy has the potential to revolutionize thermofluidic sciences by providing meaningful physical descriptors and their inextricable relationships in the context of heat and mass transfer performance.

## 2 Results

### 2.1 Artificial Intelligence‐Enabled Computer Vision

**Figure 1:**
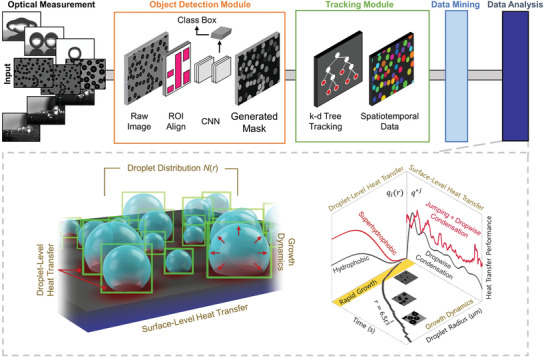
Vision‐based deep learning framework consisting of object detection, tracking, and data processing modules. High‐resolution images are collected using high‐speed optical imaging to create a large input image dataset. The images are then processed through an object detection module (orange frame) consisting of an integrating Mask R‐CNN with a ResNet‐101 backbone where objects are automatically detected to generate instance‐segmented masks. The objects in the masks are then linked and tracked using a tracking module (green frame) based on TrackPy employing *k*‐dimensional tree algorithms for object linkage. The tracked results are post‐processed using a data processing module to extract multi‐level physical descriptors such as droplet growth rate, heat flux, and droplet distributions (gray dotted frame). Images shown in the top left region contain droplets which range in size scale from 10 µm to 1 mm. Images are shown as exemplary datasets and are qualitative descriptors. Schematics are not to scale.

We develop a modular framework that consists of AI‐enabled object detection, object tracking, and data processing modules to extract meaningful features from image datasets. The object detection module (**Figure** **1**) uses high‐resolution droplet images and first passes them through a custom‐trained instance segmentation model (Mask R‐CNN) where droplet masks are assigned with unique identifiers (IDs). At this stage, the model records primitive spatial features such as equivalent diameter, pixel‐wise area, eccentricity, orientation, solidity, and location. Following the object detection module, a separate object tracking module takes the detected masks and passes them through a tracking toolkit (TrackPy) where the identified spatial features are used as parameters for tracking via the *k*‐dimensional (*k*‐d) tree algorithm. During the droplet tracking process, potential errors are manually identified and corrected using a documented graphical user interface (GUI).^[^ ^47^ ^]^ The model accuracy from the object detection/tracking is validated by testing evaluation metrics such as recall, precision, accuracy, F1‐score, and mean average pixel error (MAPE) (see Section S1 and Figure S1, Supporting Information).^[^ ^48^ ^]^ Last, a data processing module post processes the datasets to extract higher‐level features such as individual droplet growth rates and temporal heat transfer, with visualization enabled with Matlab. We mitigate Mask R‐CNN prediction fluctuations (± 300 nm) when processing the data by employing the moving average method over 10 frames. The extracted features are further treated by implementing in‐house algorithms to acquire explicit experimental results. First, the algorithms filter peripheral droplets to avoid anomalies caused by clipped droplets (see Figure S2, Supporting Information). Furthermore, we isolate condensate growth from coalescence‐induced droplet growth by setting model configurations to identify merging events with new droplet IDs. For example, if droplets having IDs of 1 and 2 coalesce, the model identifies the merged droplet with a new ID.

### 2.2 Acquisition of the High Spatio‐Temporal Resolution Heat Flux

To identify parameters that govern the dropwise condensation, the framework was first used to map the spatio‐temporal heat transfer by tracking individual droplet statistics from live images. The heat transfer rate per individual droplet *q_i_* is a crucial parameter that is classically challenging to acquire using conventional methods. The *q*_i_ can be computed with an energy balance ( $q_{i}={\dot{m}}_{i}\;h_{\mathrm{fg}}=\rho_{w}\;h_{\mathrm{fg}}dV_{i}/dt$), where the condensate mass accumulation rate is related to the growth rate of an individual droplet by^[^ ^16^, ^19^ ^]^

(1) $$q_{i}^{j}=\;\pi \rho_{w}h_{\mathrm{fg}}{\left(1-\cos\theta_{a}\right)}^{2}\left(2+\cos\theta_{a}\right){r_{i}^{j}}^{2}\frac{dr_{i}^{j}}{dt}\;$$

where *h*_fg_ is the latent heat of vaporization, *ρ*_w_ is the liquid density, *r_i_* is the individual droplet radius, d*r*_i_/d*t* is the individual droplet growth rate, and *θ*_a_ is the apparent advancing contact angle. Therefore, the droplet‐level, local heat flux $q_{i}^{j\prime \prime }$ can be calculated by normalizing the droplet heat transfer rate $q_{i}^{j}$ by the droplet base area $A_{b,i}^{j}=\pi r_{b,i}^{j^{2}}$

(2) $$q_{i}^{j\prime \prime }=\frac{q_{i}^{j}}{\pi r_{b,i}^{j^{2}}}$$

where *r*_b,i_ is estimated from optical side‐view droplet images taken from a microgoniometer. By integrating the heat transfer rate of individual droplets $q_{i}^{j}$ at an instantaneous time *j*, the instantaneous area‐averaged heat flux $q^{\prime \prime j}$ can be obtained

(3) $$q^{\prime \prime j}=\frac{\sum_{i=1}^{n}q_{i}^{j}}{A_{s}}\;\;$$

where *A*_s_ is the total surface area, and *n* is the total number of droplets at that particular time step. Then the time‐averaged heat flux becomes

(4) $$\bar{q^{\prime \prime }}=\frac{\sum_{j=1}^{Z}q^{\prime \prime j}}{Z}\;\;$$

where *Z* is the total number of time steps. We apply our models to two experimental surfaces: i) a functionalized *hydrophobic* (HP) *surface* (*θ*_a_ = 118°) and ii) a nanotextured *superhydrophobic* (SHP) *surface* (*θ*_a_ = 169°). For each surface, images are captured over a 10 min measurement period, which is equivalent to roughly 220 000 instances per dataset.

### 2.3 Deep Learning Analysis of Dropwise Condensation on a Hydrophobic Surface

**Figure 2:**
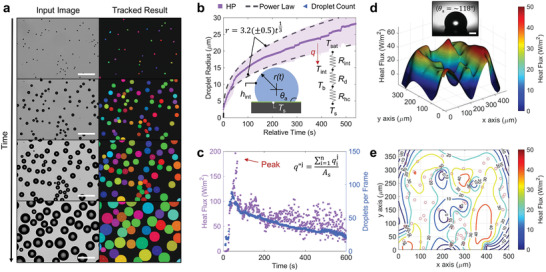
Deep learning microscale droplet‐resolved heat flux mapping. a) Time‐lapse images of raw and tracked results for dropwise condensation on the HP surface. Scale bar represents 100 µm in all images with the time interval between each frame being ≈30 s. The gravitational vector points into the page. b) Time evolution of the average droplet diameter. The dotted lines represent the power law fit. Rapid growth at early stages (*r* < 10 µm) is due to the relative scale of the variable thermal resistances illustrated in the inset. Shaded areas represent the standard deviation of the collected data. c) Instantaneous area‐averaged heat flux $q^{\prime \prime }j$ as a function of time, showing an optimum exists which is closely related to the droplet population plotted in blue (right axis). Spatially resolved heat flux $q_{i}^{\prime \prime j}$ for multiple droplets as represented by d) 3D surface and e) 2D contour plots. The circles in the contour plot mark the centroid locations of individual droplets. The inset in (d) shows an optical image of the apparent advancing contact angle (*θ*_a_ = 118°) of a water droplet residing on the HP surface. Inset scale bar: 100 µm.

The hydrophobicity of the tested HP surface facilitates discrete droplet nucleation, growth, and coalescence, as shown in the time‐lapse tracked results of **Figure** **2**a and Movie S1 (Supporting Information). The HP surface did not show any droplet jumping phenomena. The horizontal orientation of the HP surface in this experiment prevents droplets from being removed via gravitational sweeping. As a result, droplets continuously grow from droplet nucleation (relative time = 0 s) until coalescence (Figure 2b). The droplet growth follows the power‐law exponent model *r* = *αt* ^*β*^,^[^ ^16^, ^49^, ^50^ ^]^ where the exponent *β* = 1/3 ^[^ ^16^ ^]^ adequately fits the experimental data when *α*  =  3.2 ± 0.5. The relatively high growth rates approaching d*r*/d*t* = 0.2 μms^–1^ during the early stages (*r* ≤ 10 µm) of condensation gradually decrease to (d*r*/d*t* = 0.08 μms^–1^), which can be understood by the increase in heat conduction resistance as droplets enlarge (*r* > 10 µm). A detailed thermal resistance analysis is shown in Section S2 and Figure S3 (Supporting Information). Interestingly, the high growth rate at *r*  =  9 µm in Figure 2b contributes to the highest, instantaneous area‐averaged heat flux $q^{\prime \prime }j$ showing a peak of ≈200 Wm^–2^ at *t* = 24 s in Figure 2c along with the maximum droplet population. Therefore, the absence of droplet removal mechanisms inhibits new droplet formation, leading to the gradual decrease in droplet population and area‐averaged heat flux $q^{\prime \prime }j$ from *t* = 24–600 s (Figure 2c).

It is essential to map individual droplet‐resolved heat flux $q_{i}^{\prime \prime j}$ in order to understand the condensation dynamics and mechanisms that govern the collective assessment of the area‐averaged heat flux $q^{\prime \prime }j$ Here, the droplet statistics are converted to spatio‐temporal heat flux $q_{i}^{j\prime \prime }$ information using Equation (2) where $r_{b,i}^{j}\;\approx \;0.92r_{i}^{j}$ is estimated via optical side view droplet images taken from a microgoniometer and shown in the inset of Figure 2d. Figure 2d,e shows a representative real‐time surface and contour heat flux map at *t* = 25 s. A bi‐harmonic spline interpolation method is used to interpolate the irregularly spaced data points.^[^ ^51^ ^]^ In agreement with the overall surface heat flux curve shown in Figure 2c, higher heat fluxes at the early stages *t* < 24 s are clearly shown in our time‐lapse droplet‐level heat flux mapping video (Movie S2, Supporting Information).

### 2.4 Deep Learning Analysis of Droplet Jumping on Superhydrophobic Surfaces

**Figure 3:**
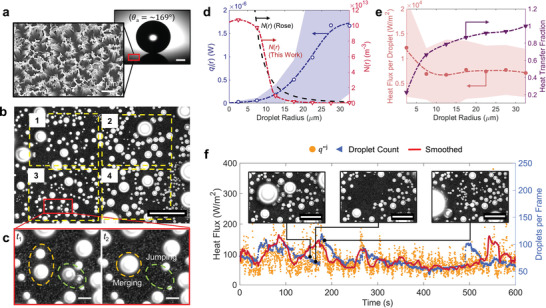
Deep learning heat transfer analysis of superhydrophobic surfaces. a) Scanning electron microscopy (SEM) image of the superhydrophobic (SHP) Si nanowire surface. Inset: optical image of a water droplet sitting on top of the SHP surface. Scale bar in the inset represents 100 µm. b) Top‐view optical microscopy images showing dropwise condensation on the SHP surface. The gravitational vector points into the page. To capture the stochastically varying nature of droplet jumping, we divide the image into sections, referred to as SHP 1, 2, 3, and 4. The scale bar represents 50 µm. c) Optical images showing both droplet merging (yellow) and droplet jumping (green) events. The scale bar represents 5 µm. d) Size‐dependent heat transfer rate per droplet *q_i_* (*r*) and droplet size distribution *N*(*r*) as a function of droplet radius. The black dashed line represents Rose model. Shaded areas represent one standard deviation. e) Experimentally measured heat flux and cumulative fraction of the overall heat transfer as a function of droplet radius. The heat flux was calculated using the developed deep learning methodology by normalizing the heat transfer per droplet by the droplet–surface contact area. Shaded areas represent one standard deviation. f) Instantaneous area‐averaged heat flux $q^{\prime \prime }j$ (orange) shows matching peaks with droplets (blue) per frame. Insets: optical images of droplets before a jumping event, instantly after a jumping event, and when new droplets start forming. For better visualization, the raw $q^{\prime \prime }j$ data points are smoothed using the LOESS function (red).

Heat transfer can be further improved by facilitating the rapid removal of droplets at smaller length scales with the use of SHP surfaces (**Figure** **3**a,b) that enable coalescence‐induced droplet jumping (Figure 3c).^[^ ^11^ ^]^ Our deep learning results reveal that the overall heat transfer is governed by the combination of size‐dependent heat transfer rate per droplet *q_i_* (*r*) and the time‐averaged droplet distribution *N*(*r*). Figure 3d plots *q_i_* (*r*) using Equation (1) and *N*(*r*) as a function of droplet radius^[^ ^52^ ^]^

(5) $$N\left(r\right)=\;\frac{\sum_{j\;=\;1}^{Z}\sum_{i\;=\;1}^{n}\left[r-\frac{1}{2}\Delta r<\;r_{i}^{j}<r+\frac{1}{2}\Delta r\right]}{A_{s}\Delta rZ}\;$$

where *Z* is the total number of time steps and Δ*r* is the distribution bin width of 5 µm. As evident in Figure 3d and Equation (1), *q_i_* (*r*) is governed by droplet size and increases as the droplet grows. Our experimental *N*(*r*) matches well with the theoretic model proposed by Rose^[^ ^53^ ^]^ and is highly skewed toward small (*r* < 12.5 µm) droplets, suggesting a tradeoff between *q_i_* (*r*) and *N*(*r*).

Figure 3e elucidates the tradeoff by plotting the heat transfer fraction *f*, defined as the cumulative heat rate normalized by the total heat transfer rate (*q*_*i*, cumul_ /$\;{\bar{q}}_{i}$) as a function of droplet radius

(6) $$f\;=\;\;\frac{q_{i,\;\mathrm{cumul}}}{{\bar{q}}_{i}}=\frac{\sum_{i=1}^{Z}q_{i}\left(r_{1}\right)}{\sum_{i=1}^{Z}q_{i}\left(r_{2}\right)}\;\;$$

where 0 < *r*_1_ < *r* and 0 < *r*_2_ < *r*_max_. The heat transfer fraction is *f* ≈ 0.8 when the droplet radius is 12.5 µm, meaning that the integrated heat transfer of droplets with radii less than 12.5 µm contribute to 80% of the total heat transfer, despite having low *q_i_* (*r*) as shown in Figure 3d. We note that in contrast to the low *q_i_* (*r*) of emergent droplets, the local heat flux at an instantaneous time *j* per droplet $q_{i}^{\prime \prime j}$ is maximized during initial stages due to the minimal basal area $A_{b,i}^{j}$ (Figure 3e). However, $q_{i}^{\prime \prime j}$ becomes invariant with droplet radius after *r* > 7.5 µm as the increase in $A_{b,i}^{j}$ and *q_i_* (*r*) counterbalance each other. All analyzed experiments suggest that the generation of large populations of fresh droplets is favorable for maintaining high surface‐ and droplet‐level heat transfer performance.

Figure 3f supports our findings by qualitatively visualizing the relationship between area‐averaged heat flux at an instantaneous time *j*, $q^{\prime \prime }j$, and droplet population. Figure 3f shows that the SHP surface maintains a stable $q^{\prime \prime }j$ throughout the measurement period when compared to the HP surface (Figure 2c). The constant heat transfer performance is attributed to the capability of the SHP surface to remove droplets via droplet jumping. In particular, we observe numerous cases where binary droplet–droplet coalescence events trigger serial interactions with adjacent droplets prior to jumping, leaving large dry patches for new droplets to nucleate (Figure 3f, inset). In agreement with this observation, we report the existence of heat flux surges when dry areas fill with dense populations of new droplets. Similar findings are reflected in the real‐time surface and contour plots provided in Movie S3 (Supporting Information). The temporal area‐averaged heat flux $q^{\prime \prime }j$ is smoothed using a LOESS regression to clearly visualize the consistent heat fluxes over the time (Figure 3f) despite fluctuations stemming from dynamic changes in droplet sizes and corresponding heat transfer rates.^[^ ^54^ ^]^

### 2.5 Deep Learning Analysis of Condensation Statistics

**Figure 4:**
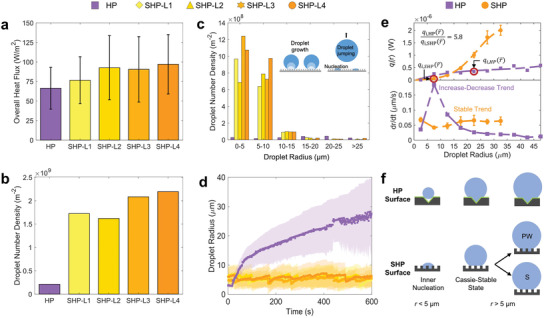
Deep learning characterization of condensation dynamics. a) Time‐averaged overall surface heat flux $\bar{q^{\prime \prime }}$ averaged over the measurement time, showing higher heat fluxes for the SHP surface. The error bar represents one standard deviation. b) Droplet number density at different locations and surfaces. c) Histogram of the droplet size distribution averaged for a 10 min condensation interval. Inset: schematic of a droplet growth, jumping, and nucleation cycle on the SHP surface. d) Time‐averaged droplet radius as a function of time. The average droplet size continuously increases for the HP surface as droplets continue to coalesce and grow. The SHP surface is able to maintain a relatively consistent droplet size due to jumping droplet removal. e) Comparison of individual droplet heat transfer rate for the HP and SHP surfaces revealing that the droplet‐level heat transfer increases at different rates as the droplet radius increases. The heat transfer rate for the average droplet radius $q_{i}\left(\bar{r}\right)$ for both surfaces is circled in red. The bottom subplot suggests that the difference in $q_{i}\left(\bar{r}\right)$ is due to the distinctive growth rates d*r*/d*t*, where the HP surface exhibits an increase‐decrease trend while the SHP surface remains steady. f) Schematics of the growth trends on the HP and SHP surfaces illustrating the evolution of the droplet wetting based on nucleation (*r* < 5 µm) and growth (*r* > 5 µm) stages. The mixture of partial wetting (PW) and suspended (S) droplets compensates for the low growth rate on the SHP surface.

To quantify how the combination of size‐dependent heat transfer rate per droplet *q_i_* (*r*) and the droplet density affect the overall heat transfer performance, we used the deep learning methodology to compare droplet statistics for the HP and SHP surface. Unlike the merging‐dominated droplet interactions that occur on the HP surface, the stochastically cyclic droplet interactions on the SHP surface are more prone to exhibiting location‐dependent heat transfer variation. To verify location‐independence, we analyze four different locations on the SHP surface, as illustrated in Figure 3b. Our results show that sustainable dropwise condensation can be achieved on the SHP surface regardless of location, attaining a 35% higher time‐averaged heat flux $\bar{q^{\prime \prime }}$ of 89 Wm^–2^ when compared to 66 Wm^–2^ on the HP surface (**Figure** **4**a). The droplet number density in the field of view (560 µm × 370 µm) for the SHP surface is ≈800% higher when compared to the HP surface (Figure 4b), with > 90% of the droplets having *r* < 10 µm (Figure 4c). Figure 4d elucidates the effectiveness of the SHP surface at maintaining small droplet sizes over time, where a time‐average droplet radius $\bar{r}$ ≈ 22 µm and ≈7 µm is shown for the HP and SHP surfaces, respectively. Interestingly, we show that the drastic droplet density discrepancy for the HP and SHP surfaces (Figure 4b–d) is disproportionately reflected on the overall heat transfer performance (Figure 4a).

To investigate the mismatch between droplet statistics and heat transfer performance, we used the deep learning method to analyze and compare the heat transfer rate per average droplet radius $q_{i}\left(\bar{r}\right)$ of the HP and SHP surfaces (Figure 4e). The distinctive heat transfer rate per individual droplet curves shown in Figure 4e are a result of surface‐dependent droplet growth mechanisms. Figure 4e (bottom plot) quantifies d*r*/d*t* as a function of droplet radius, showing distinct droplet growth trends for the HP and SHP surfaces, which is caused by the interchanging surface‐droplet thermal contact resistances (Figure 4f) explained in Section S2 (Figure S4) (Supporting Information) and reported elsewhere.^[^ ^16^ ^]^ Due to these surface‐dependent droplet growth mechanisms, it becomes imperative to understand which droplet size represents the total heat transfer performance the most. By assuming that the average droplet radius is the characteristic droplet size for each surface, the overall heat flux can be explicitly calculated by $\bar{q^{\prime \prime }}=\;Pq_{i}\left(\bar{r}\right)/A_{s}$ (see Section S3 in the Supporting Information for model development), where *P* is the number of droplets during the entire experimental period. Based on this calculation, we estimate that the SHP surface would require ≈6 times higher droplet number density to equate the $\bar{q^{\prime \prime }}$ to the HP surface (Figure 4e). Experimentally, we observe that the SHP has ≈9 times higher droplet population density than the HP surface (Figure 4b), such that the increase matches the increase in the overall heat flux shown in Figure 4a.

## 3 Discussion

The deep learning, vision‐based framework we propose enables data‐centric thinking and a comprehensive approach toward optimizing surface designs for condensation heat and mass transfer. Surface design rules for stable dropwise condensation are currently empirical due to the weak mechanistic connection between heat transfer and the large time‐ and length‐scale bandwidth of droplet statistics. The ambiguity between the heat transfer and droplet statistics stems from the difficulty in measuring heat flux from condensation experiments operating in low superheats. As mentioned in past studies,^[^ ^55^ ^]^ characterizing the heat flux from a condensing surface has posed a challenge for researchers for decades due to the small temperature differences (Δ*T*  =  1−3 *K*) that are measured, which are usually within the uncertainty of the thermocouples used for the measurement. The natural difficulty of extracting instance‐level features from surfaces with large droplet desnsities has been an additional bottleneck for researchers. The capability of our framework to study the heat rate per individual droplet *q_i_* (*r*), not only offers a solution to both of these challenges, but also reveals the essential requirement to co‐design the size‐dependent heat transfer rate per droplet *q_i_* (*r*) and droplet number density to maximize the overall heat transfer performance. That is, instead of designing surfaces to target larger surface coverage of small droplets, we show that droplets should grow to an optimal size while achieving maximum allowable number density, which is potentially achievable with spatial control of droplet nucleation as well as apparent local contact angle.

**Figure 5:**
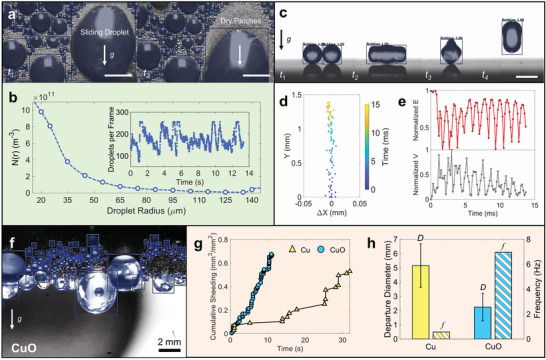
Deployment of the deep learning framework for novel tasks. The proposed framework was expanded to address visual tasks in alternate condensation applications. a) Time‐lapse front‐view images of sliding ethanol condensate droplets on a lubricant‐infused surface (LIS). The tracked droplets overlay the image with added bounding boxes for clearer visualization. The scale bar represents 300 µm. b) Droplet distribution for the process shown in (a) as calculated using Equation (5) and showing the statistics gathered over 141838 instances. Inset: Number of droplets per frame was a function of time, showing the higher fluctuation on the LIS surface when compared to the HP and SHP surfaces due to active shedding dynamics. c) Time‐lapse side‐view images of coalescence induced water droplet jumping. The scale‐bar represents 800 µm. d) Vertical droplet jumping trajectories. e) Normalized eccentricity and velocity evolution of the jumping droplet shown in (c). f) Instantaneous image with tracking results for external condensation of steam on a horizontal SHP tube sample. g) Cumulative departure volume per unit area shows that the CuO SHP surface sheds droplets at a higher rate than the HP Cu surface. h) Average departure diameter and shedding frequency of both Cu and CuO surfaces where filled colored bars represent the departure diameter (left axis) and hatched bars represent shedding frequency (right axis). The error bars represent one standard deviation.

In addition to providing a deep and quantitative explanation about droplet condensation mechanisms, our framework converging computer vision and data science is a powerful tool for the potential application to other image‐based thermofluidic measurements (**Figure** **5**). The primary advantage of employing teachable models is their capability to learn and adjust with additional data. By implementing our framework, we showcase several other configurations, including shedding dynamics during condensation on a lubricant‐infused surface (LIS; Figure 5a,b), side‐view droplet jumping dynamics (Figure 5c–e), and shedding dynamics during external tube condensation (Figure 5f–h). Figure 5a,b demonstrate the high‐fidelity measurement of droplet distributions despite the co‐existence of large and small droplets, where large droplets are shed due to gravity. In Figure 5c–e, we demonstrate a means for our deep learning method to save hundreds of hours of manual labor for analyzing side‐view jumping droplet characteristics, such as droplet trajectory, velocity, or shape. In Figure 5f–h, we use our deep learning methodology to compare shedding characteristics of textured and nontextured copper tubes by extracting droplet statistics, departure diameters, and their shedding frequency. Further details are provided in Section S4 (Supporting Information). Our demonstrations synergistically combine intelligent vision and data mining, which enables the experimental acquisition of high‐resolution spatio‐temporal information reflecting condensation mechanisms. With the advances reported here and recent breakthroughs in deep learning‐based visualization strategies,^[^ ^56^ ^]^ we expect accelerated progress in pushing the knowledge boundaries of thermofluidic science by enabling the rapid and accurate analysis of massive image datasets.

## 4 Experimental Section

### Model Training

Pixel‐wise masks were created from image inputs (Figure S1a–d, Supporting Information) by using Mask R‐CNN to further identify object information. A custom Mask R‐CNN model was trained on a custom‐built image inventory consisting of thousands of droplet images acquired over years of condensation experiments conducted in various lighting, surfaces, and orientations. Image annotation, the process of manually labeling images to train the model, was performed by a group of trained annotators over the course of six months using commercial annotation software (Supervisely, San Jose, CA, USA). The generalizability of the annotated images was maximized through data augmentation techniques, where the original data were transformed into new, increased, and slightly modified versions. Further details of the data augmentation process were discussed in a previous work.^[^ ^32^ ^]^ 80% (1968 images) and 20% (492 images) of the augmented dataset images were used for training and testing, respectively. The Mask R‐CNN model used for this study was trained for 100 epochs using stochastic gradient descent with a learning rate of 0.0012 and momentum of 0.9. To increase detection accuracy, the results of two models were averaged with tests of loss of ≈0.1 and 0.11 at epochs 98 and 100, respectively (Figure S1e, Supporting Information).

### Model Evaluation

The model performance was evaluated by analyzing multiple metrics such as recall, precision, accuracy, F1‐score, and mean average pixel error (MAPE; Section S1 and Figure S1f,g, Supporting Information). The model demonstrated exceptional detection capabilities with values > 96% for conventional metrics and a low MAPE of 3%.

### Top View Optical Microscopy of Water Vapor Condensation

Top view condensation experiments were conducted on a customized top‐view optical microscopy setup implemented with a temperature controllable cold‐stage. A high‐resolution camera (DS‐Qi2, Nikon) was attached to an upright microscope (Eclipse LV100, Nikon) equipped with a 20X (TU Plan Fluor EPI, Nikon) objective lens and records condensing droplet images at 1–10 fps. Test samples were horizontally mounted onto a cold‐stage (PE‐120, Linkam) and the sample surface temperature was set to 1 ± 0.5 °C for condensation experiments. All the experiments were performed in ambient laboratory temperature. Relative humidity of environment was controlled by a commercial humidifier and the humidity level was recorded and logged by a temperature and relative humidity transmitter (HX93BDV0, Omega). A more detailed description of the experimental setup could be found in previous works.^[^ ^57^, ^58^ ^]^

### Front View Optical Imaging of Ethanol Condensation Dynamics

To characterize the low surface tension liquid droplet size measurement and distribution (Figure 5a), dropwise condensation experiments of ethanol (200 proof, ≥99.5%, liquid–vapor surface tension 23.03 mN m^–1^ at 10 °C, Sigma‐Aldrich, CAS No. 64‐17‐5) on the flat LIS sample were conducted following procedure described in a previously reported work.^[^ ^24^ ^]^ A digital single‐lens reflex (DSLR) camera (Canon 6D) mounted with objective lenses (Nikon, Plan Fluor) of 20× magnification was employed for imaging.

### Front View Optical High‐Speed Imaging of Water Droplet Coalescence Induced Jumping

Front view imaging of droplet jumping was captured by employing a microdroplet dispensing and manipulating system. (Figure 5c).^[^ ^13^ ^]^ A high‐speed camera (Phantom v711, Vision Research) was interfaced with a piezoelectric microgoniometer (MCA‐3, Kyowa Interface Science). The piezoelectric dispenser was frequency‐controlled and dispensed monodisperse microscale droplets having radii of ≈20 µm. Droplet dispensing conditions were 7 V, 100–200 Hz. The addition of the dispensed microdroplets showed negligible effects on target droplet coalescence and jumping dynamics.^[^ ^13^ ^]^ Imaging with a ≈25X magnification lens was conducted with a capture rate of 13 000 fps. An LED source (TSPA22×8, AITECSYSTEM) supplied illumination. Experiments were conducted in ambient conditions with a temperature of 24 ± 1 °C and relative humidity of 40 ± 5%.

### Front View Optical Imaging of Steam Condensation Dynamics on a Horizontal Tube

The experimental setup for external tube condensation consisted of an environmentally controlled chamber having an internal diameter of 0.305 m and length of 0.559 m. In brief, the chamber was initially evacuated to *P*_v_ = ≈2 Pa to remove the noncondensable gases. During the pump down process, steam was generated in a separate smaller stainless‐steel pressure vessel. Valves were incorporated into all fluid lines to control flow in and out of the vapor generator. A chilled water flow loop was used to cool the tube samples to promote condensation on the tube outer surface. The water flow rate was measured using an electromagnetic flowmeter and the inlet–outlet temperatures into the test section were measured using class A resistance temperature detectors (RTDs). Visual recordings of the condensation process were captured with a high‐speed camera (Phantom v7.1, Vision Research) and a digital SLR camera (Pentax K‐50), which were placed in line with two different viewports. Details regarding the experimental setup and procedure could be found elsewhere.^[^ ^59^, ^60^ ^]^

### Fabrication of the Hydrophobic Silicon Surface

To fabricate the hydrophobic (HP) silicon surface, a polished silicon wafer (<100>, 350 µm, 444, University Wafer) was functionalized with (heptadecafluoro‐1,1,2,2‐tetrahydrodecyl) trimethoxysilane (HTMS, CAS No. 83048‐65‐1, SIH5841.5, Gelest) via chemical vapor deposition (CVD).^[^ ^61^ ^]^ The silicon wafer samples were placed in a beaker container with a vial of 1 mL of HTMS‐toluene mixture solution (5% v/v). An aluminum foil lid was placed on top to seal the beaker, followed by heating in an atmospheric pressure oven (Thermo Scientific BF51732C‐1) at 85±1 °C for 3 h. The advancing contact angle of the hydrophobic silicon wafer surface was measured to be 118° as shown in Figure 2d.

### Fabrication of the Superhydrophobic Surface

A polished silicon wafer (P/B type, <100>) was sonicated in an acetone solution followed by soliciting with isopropyl alcohol (IPA), deionized (DI) water, and IPA for 5 min, respectively. The samples were then dried under a clean nitrogen gas flow. The cleaned silicon wafer was then immersed in a composite solution of silver nitrate (AgNO_3_), DI water, and hydrogen fluoride (HF) for 2 min to deposit Ag nanoparticles on the silicon surface as an etching mask. Then, the wafer was immersed into another composite solution of DI water, HF, and hydrogen peroxide (H_2_O_2_) for metal‐assisted electrodeless etching for 5 min to create silicon nanowires. Silver dendrites on the surfaces were removed by immersing the wafer into a solution of nitric acid (HNO_3_) solution followed by immersion in DI water and drying on a hot plate. The fabricated silicon nanowire surface had nanowire diameters of 100 nm and lengths of ≈2 µm, verified using an SEM characterization technique (Hitachi S‐4800). The silicon nanowire surface was then functionalized to become superhydrophobic using the identical functionalization method described for the HP surface. The apparent advancing contact angle of water droplets on the SHP silicon wafer surface was 169°.

### Fabrication of the Superhydrophobic CuO Surface and LIS Surface

The fabrication steps of LIS consisted of surface cleaning, copper oxide (CuO) nanostructure fabrication, hydrophobic functionalization, and lubricant impregnation.^[^ ^59^ ^]^ Initially, the Cu plates were cleaned by sonicating them sequentially in acetone, ethanol, and isopropyl alcohol for 10 min at room temperature. After cleaning, the surfaces were rinsed with deionized water and dried with nitrogen. Subsequently, the samples were immersed in 2.0 m diluted hydrochloric acid for 10 min to remove native oxides that were present on the surface, rinsed with deionized water, and thereafter dried with nitrogen. The CuO nanostructure fabrication was carried out by immersing the cleaned copper samples for 25 min into a pool of alkaline solution maintained at 90 ± 3 °C. The alkaline solution consisted of a mixture of 3.75 wt% NaClO_2_, 5 wt% NaOH, 10 wt% Na_3_PO_4_·12H_2_O, and 100 wt% deionized water. Subsequently, the CuO nanostructures were functionalized by chemical vapor deposition of heptadecafluorodecyltrimethoxy‐silane at atmospheric pressure. The functionalization was carried out by first placing the samples and 5 mL of HTMS‐toluene solution (5% v/v) in a sealed container. The container was placed inside a furnace at 90 °C for 3 h resulting in the deposition of a silane monolayer of HTMS molecules on the sample surfaces. Finally, to develop LIS, the functionalized CuO nanostructured samples were immersed into a lubricant bath (perfluorinated vacuum grade oil Krytox VPF 1525) for 10 min. Subsequently, the samples were removed from the lubricant pool and left vertically in ambient temperature for 24 h to allow excess lubricant to be drained by gravity.

## Conflict of Interest

The authors declare no conflict of interest.

## Supporting information

Supporting Information

Supplemental Movie 1

Supplemental Movie 2

Supplemental Movie 3

## Data Availability

The data that support the findings of this study are available from the corresponding author upon reasonable request.
