# Supplementary material for: A Deep Learning Perspective on Dropwise Condensation
Source: Adv Sci (Weinh). 2021 Sep 24;8(22):2101794. doi: 10.1002/advs.202101794 (PMC8596129; doi:10.1002/advs.202101794)
Supplement: Supplementary file 1 — Supporting Information [file ADVS-8-2101794-s003.pdf]

© 2021 Wiley-VCH GmbH

ADVANCED SCIENCE
Open Access

Supporting Information

for *Adv. Sci.,* DOI: 10.1002/advs.202101794

A Deep Learning Perspective on Dropwise Condensation

*Youngjoon Suh, Jonggyu Lee, Peter Simadiris, Xiao Yan, Soumyadip Sett, Longnan Li, Kazi Fazle Rabbi, Nenad Miljkovic, Yoonjin Won**

Supporting Information

A Deep Learning Perspective on Dropwise Condensation

Youngjoon Suh, Jonggyu Lee, Peter Simadiris, Xiao Yan, Soumyadip Sett, Longnan Li, Kazi Fazle Rabbi, Nenad Miljkovic, Yoonjin Won

*Corresponding author. Email: won@uci.edu and nmiljkov@illinois.edu

This PDF file includes:

Section S1: Model configuration
Section S2: Droplet growth mechanism
Section S3: Heat transfer rate per average droplet radius
Section S4: Tunable framework
Figure S1. Object detection module.
Figure S2. Eliminating peripheral droplet effects.
Figure S3. Thermal resistance breakdown of a droplet condensing on a hydrophobic surface as a function of droplet diameter.
Figure S4. Thermal circuit schematic of a superhydrophobic surface.
Supporting information references (1-11)

Other Supporting Information for this manuscript include the following:

Movie S1. Time-lapsed tracked results.
Movie S2. Surface and contour heat flux mapping of hydrophobic surface.
Movie S3. Surface and contour heat flux mapping of superhydrophobic surface.

Section S1. Model configuration

Mask R-CNN accuracy. A script is developed to determine the performance of the model using a commercial programming software (MATLAB). The script binarizes the predicted dataset and ground truth dataset, then checks if the predicted dataset pixels correspond with the ground truth pixels. A true condition occurs when pixels are equal to each other, similarly a false condition occurs when the pixels do not equal each other. A positive condition is when the model detects an instance, whether it is true or false. A negative condition is when the model does not detect an object.[1] That is, true and false positive (TP/FP) is defined as the number of positives that were correct/incorrect and is similarly defined for true and false negatives (TN/FN). These conditions are summed across the dataset and used to determine the performance of the model.

The performance metrics used to validate the model were accuracy, recall, precision, and F1 score, and pixel positivity. Accuracy is the ratio of the sum of true positive instances to the overall positive instances, showing how often the model predicts a pixel correctly:[2]

$$Accuracy = \frac{TP + TN}{TP + TN + FN + FP} \quad (S1)$$

Recall is the ratio of true positive instances to the sum of overall true positive instances and false negative instances, representing how often the model predicts a true positive instance correctly with respect to all observed positive instances:[2]

$$Recall = \frac{TP}{TP + FN} \quad (S2)$$

Precision is the ratio of true positive instances to the sum of all observed true instances. This describes how often a positive instance was correctly predicted compared to all positive instances:[2]

$$Precision = \frac{TP}{TP + FP} \quad (S3)$$

F1 score is the weighted average of recall and precision and does not include true negative instances. It can be used as an alternative to accuracy when true negatives are not considered significant observations.[2]

$$F1\,Score = 2 * \frac{(Precision * Recall)}{(Precision + Recall)} \quad (S4)$$

We define a metric called pixel error (PE) to characterize pixel-based prediction accuracies. PE is determined by subtracting the ground truth (GT) binary mask from the predicted binary mask (PM), then dividing by the ground truth. This results in the true negatives being removed from the binary matrix, leaving only true positives, false positives, and false negatives. Similar to recall, PE compares the ratio of predicted positive pixels to overall positive pixels. The mean absolute pixel error (MAPE) takes the average of PE as follows:

$$MAPE = \frac{1}{n}\sum_{i=1}^{n}|PE| \times 100 = \frac{1}{n}\sum_{i=1}^{n}\left|\frac{(GT - PM)}{GT}\right| \times 100 \quad (S5)$$

where n is the total number of image inputs.

Comparison with traditional computer vision methods. Our trained model outperforms traditional image segmentation techniques (e.g., adaptive and global thresholding) available on commercial software such as MATLAB and ImageJ (Figure S1f). While traditional computer vision algorithms are straightforward, computer vision engineers must decide which features are important for every given image. For example, even small light reflection variations on the surface or droplet greatly change how droplets are detected. In our case, adaptive thresholding, which minimizes uneven illumination effects by determining thresholds locally, exhibited the worst overall performance due to the combined variance of the background and droplet. Global thresholding obtained relatively better results by using a single threshold value to minimize surface reflection variations but were still susceptible to light reflection variations on the droplet. It should be further noted that unlike the binary masks that traditional computer vision techniques produce, our model generates instance-specific object masks with varying brightness intensities (Figure S1b and c) to improve the tracking process.

Peripheral droplet filter. The Mask R-CNN model employs bounding box regression to refine and predict localization boxes (i.e., bounding boxes) as shown in Figure S2.[3] Each side of the bounding box coordinate is recorded for all time frames, where $bbox_L$, $bbox_R$, $bbox_T$, $bbox_B$, represent the left, right, top, and bottom coordinate, respectively. Our filtering algorithm identifies and eliminates peripheral droplets by finding instances where $bbox_L = x_L$ or $bbox_R = x_R$ or $bbox_T = y_T$ or $bbox_B = y_B$, where x_L, x_R, y_T, y_B are the outermost left, right, top, and bottom coordinates of the image (x, y), respectively.

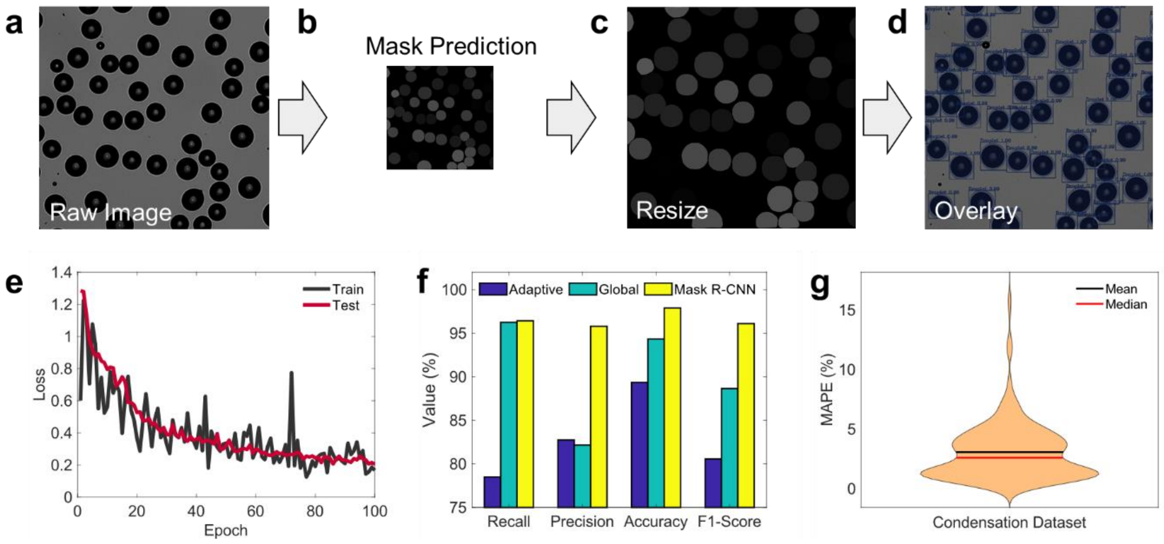

Figure S1. Object detection module. (a) Raw images are processed through a series of steps, including (b) masking, (c) resizing, and (d) final overlaying to produce the segmented image output. (e) The presented learning curves show a gradual decaying trend for both train and test loss, which implies that the model continuously is learning image features from the given dataset. Optimal checkpoints at epoch 98 and 100 were selected as object detection models. (f) Bar chart of various evaluation metrics gathered from > 500 images. The Mask R-CNN model performs better for all evaluation metrics when compared to traditional techniques that use adaptive or global thresholding values. The tracking through Mask R-CNN consistently showcases a higher accuracy. (g) The variations of the mean average pixel error (MAPE) are visualized using a violin plot.

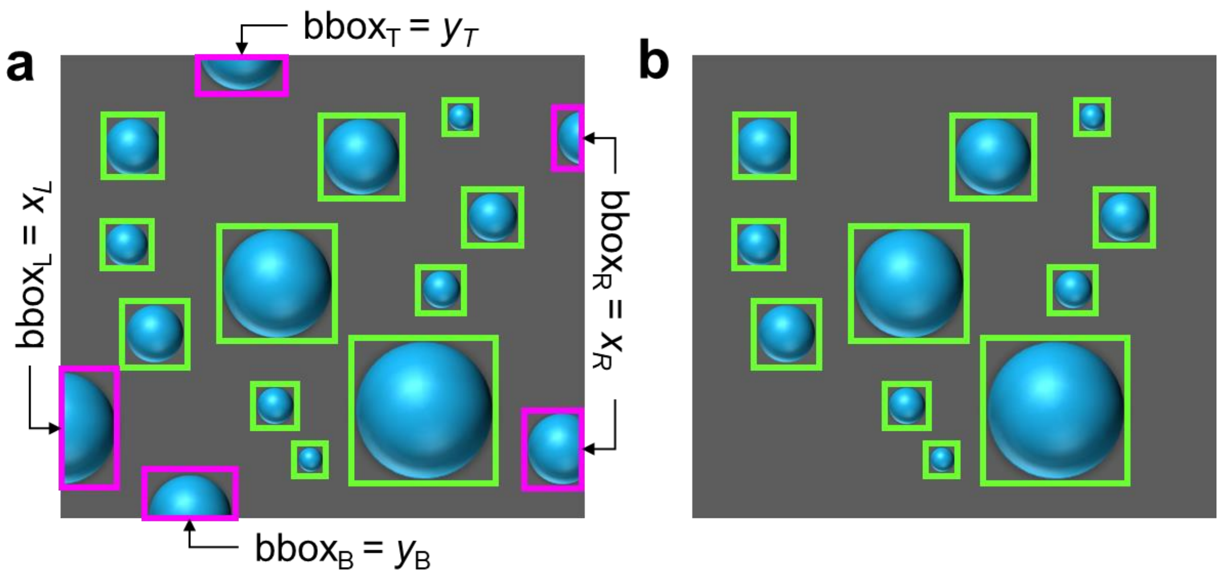

Figure S2. Eliminating peripheral droplet effects. Every droplet in the image is localized using bounding boxes (i.e., bboxes). Droplets located at the sides of the viewing window (magenta bounding boxes) do not fully reflect the growth characteristics of a full droplet (green bounding boxes) and must be filtered. The figures show an illustration of object detection results (a) before and (b) after we employ our filtering algorithm.

Section S2. Droplet growth mechanism

Thermal resistance breakdown of hydrophobic surface. The inset of Figure 2b illustrates the thermal resistance circuit of dropwise condensation on the chemically coated hydrophobic surface. The thermal resistances include the droplet liquid-vapor interface $R_{\text{int}} = 1/h_{\text{int}}2\pi r^2(1 - cos\theta_{\text{a}})$, conductive resistance through the bulk droplet $R_{\text{d}} = \theta_{\text{a}}/4\pi r k_{\text{w}} sin\theta_{\text{a}}$, and the resistance through the hydrophobic coating $R_{\text{hc}} = \delta_{\text{hc}}/\pi r^2 k_{\text{hc}} \sin^2\theta_{\text{a}}$, where h_{i} is the interfacial heat transfer coefficient in ambient conditions assumed as 0.4 $\text{MWm}^{-2}\text{K}^{-1}$, k_{w} and k_{hc} are the thermal conductivities of liquid water and the hydrophobic coating, respectively, and δ_{hc} is the thickness of the hydrophobic coating assumed as 1 μm. The breakdown of the thermal resistances plotted in Figure S3 shows that the hydrophobic coating resistance dominates heat transfer of small droplets ($2r$ < 13 μm) while conductive resistance through the bulk droplets become rapidly prominent as droplets become larger.

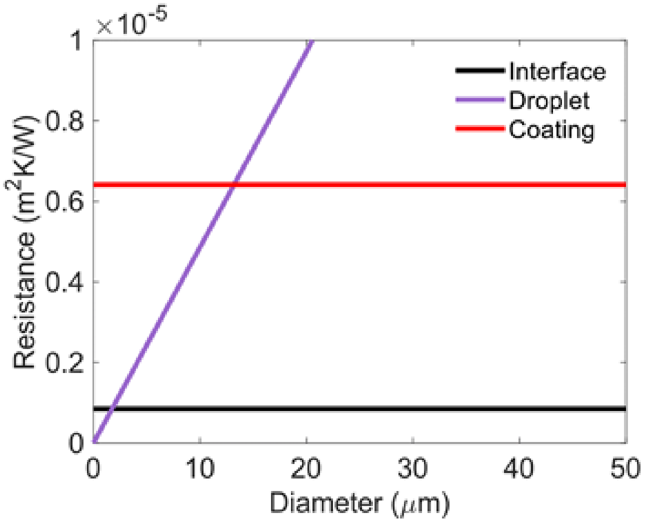

Figure S3. Thermal resistance breakdown of a droplet condensing on a hydrophobic surface as a function of droplet diameter.

Thermal resistance breakdown of droplets growing on the superhydrophobic surface. Unlike the HP surface, the SHP surface is coated with nanostructures that minimize surface-liquid adhesion forces. Figure S4 illustrates the thermal resistance circuit of a droplet suspended on the SHP surface. Due to the tortuous nature of the nanowire geometry, we lump the nanowire R_{NW} and air R_g resistances into an effective surface-liquid thermal contact resistance R_{eff} in this study.

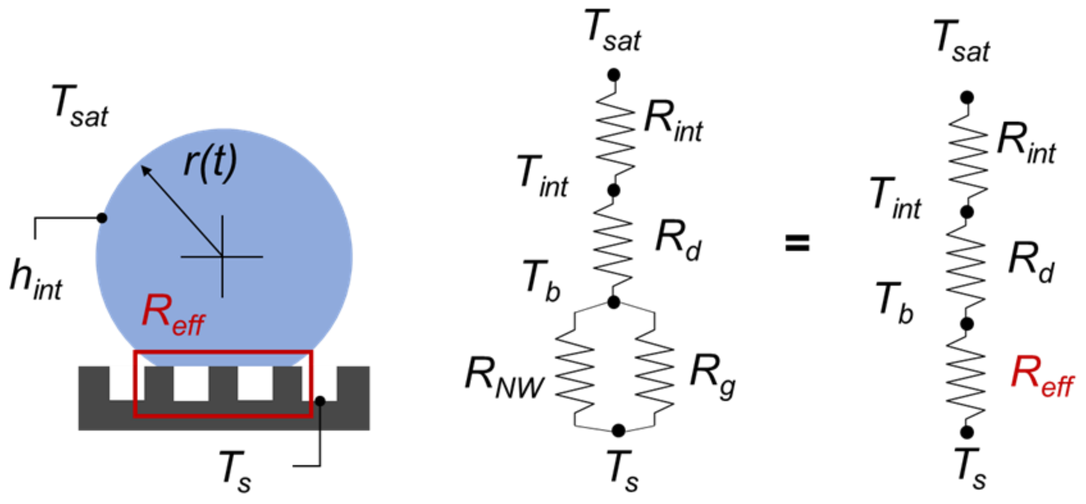

Figure S4. Thermal circuit schematic of a droplet growing due to condensation on a superhydrophobic surface.

Interchanging surface-droplet contact resistance effect on droplet growth rate. In alignment with a recent report on droplet morphology effects on growth dynamics,[4] we speculate that the distinctive growth rate dr/dt curve in Figure 4e can be explained by changes in the surface-droplet thermal contact resistance R_{eff} (Figure S4). Figure 4f illustrates how interchanging R_{eff} can influence the droplet growth of HP and SHP surfaces. When r < 5 μm, the droplet growth rates can vary depending on where the droplet initiates from. For the HP surface, the low initial droplet growth rate of ~0.03 μm suggests that the hydrophobic coating causes droplets to initiate on top of microscale defects such that an airgap exists between the droplet and the surface. This airgap hinders thermal contact, thereby increasing R_{eff}. As droplets grow, the gravity allows for the droplet to infiltrate the defect and significantly decreases R_{eff}. As noted in Figure S3, the R_{d} begins to dominate as droplet grows larger and the growth rate continuously decreases as observed in Figure 4e. Conversely, the nanostructures of the SHP surface promote droplets to form within the structure allowing for low R_{eff} when r < 5 μm. As droplet sizes exceed the characteristic length of the nanostructures, the droplet develops into a Cassie-stable state which is favorable for coalescence-induced droplet jumping. However, as the droplet size continues to increase, the droplet can either develop into a partial wetting (PW) state or remain in a suspended (S) state. The mixture of partial wetting (PW) and suspended (S) droplet states is speculated to compensate growth rates for the SHP surface.

Section S3. Heat transfer rate per average droplet radius

Model development. To provide insight into how the characteristic droplet radius effects the overall heat transfer performance, we compare the heat rate per droplet as a function of droplet radius for the HP and SHP surface (Figure 4e). The error bar represents the standard deviation acquired from location 1 – 4. The heat rate at the average droplet radius for each surface ($\bar{r}$ = ~22 µm and ~7 µm for the HP and SHP surface, respectively), is circled in red in Figure 4e. By assuming that all droplets forming on the surface is of size $r = \bar{r}$, the overall heat flux can be calculated as follows:

$$\overline{q"} = \frac{\left(\sum_{\mathrm{j}=1}^{\mathrm{Z}} q^{"\mathrm{j}}\right)}{Z} = \frac{\sum_{\mathrm{j}=1}^{\mathrm{Z}} \sum_{\mathrm{i}=1}^{\mathrm{n}} q_{\mathrm{i}}^{\mathrm{j}}(r)}{Z A_{\mathrm{s}}} = \frac{P q_{\mathrm{i}}(\bar{r})}{A_{\mathrm{s}}} \qquad (S6)$$

where Z is the total number of time steps, A_s is the total measurement domain surface area, n is the total number of droplets at a particular time step, and P is the total number of droplets during the entire experimental period. As shown in Figure 4e inset, $q_{\mathrm{i}}(\bar{r})$ is approximately 6 times higher for the HP surface when compared to the SHP surface. Equation S6 suggests that this would require P for SHP surface to be 6 times higher to achieve an equivalent $\overline{q"}$ as the HP surface.

Section S4. Tunable framework

Lubricant-infused surface condensation. Our framework first successfully analyzes the droplet population densities on a lubricant-infused surface (LIS) (Figure 5a). Recently, mico/nanotextured LISs have emerged as a robust method enabling efficient dropwise condensation of low-surface-tension fluids.[5] A recent study shows that the ultralow contact angle hysteresis and rapid droplet shedding characteristics of LISs enables a 200% condensation heat transfer performance enhancement when compared to filmwise condensation on hydrophobic surfaces.[5] However, such vigorous droplet dynamics pose a unique challenge to researchers due to the sheer number of droplets that form every second. To put the challenge into perspective, a moderate 60 fps imaging rate required to capture LIPs droplet movements would require a 5900% increase in the image dataset size compared to typical 1 fps measurements. Figure 5b shows for the first time the experimental determination of the droplet distribution $N(r)$ for LIPs surfaces, proving that the proposed framework is applicable to highly dynamic condensation environments.

Droplet jumping dynamics. Next, our framework is applied for autonomous characterization of droplet jumping dynamics (Figure S5c). Coalescence-induced droplet jumping has been a primary subject of interest over the past few years because of its potential to enhance anti-icing,[6] defrosting,[7] self-cleaning,[8] and condensation performances via passive droplet removal.[9] In order to investigate the governing parameters that affect droplet jumping behavior, researchers must incorporate both top- and side-view imaging techniques.[10] The side-view imaging analysis can consume >100 experimental trials that each require manual image analysis.[10] Figure 5c-e showcases the real-time characterization of side-view droplet trajectory (Figure 5d), morphology (Figure 5e), and velocities (Figure 5e). This shows that the automation that our framework brings towards addressing even simple image analysis tasks can potentially save hundreds of hours of unnecessary labor and research resources, which can be redirected towards other scientific purposes.

Tube condensation. We demonstrate potential insights that our framework provides for tube condensation. While many precedent studies investigate dropwise condensation on flat surfaces, tube geometries are more prevalent in industry and are thus favorable for benchmarking.[11] We analyze and compare shedding characteristics of textured and non-textured Cu surfaces by implementing custom functions to our analysis module to recognize departing droplets (Figure 5f-h). Figure 5g shows that the nanotextured CuO surface accumulates water 3 times faster than the bare Cu surface. By comparing the departing diameter and frequency (Figure 5h), we learn that the CuO surface is able to retain smaller droplet diameters while departing at elevated frequencies. Because the tube geometry is three-dimensional, future work will include the high-resolution 3D mapping of the heat transfer analysis using multiple viewports.

Supporting Information References

[1] S. Visa; B. Ramsay; A. L. Ralescu; E. Van Der Knaap, *In Proceedings of the 22nd Midwest Artificial Intelligence and Cognitive Science (MAICS) Conference,* **2011,** Cincinnati, Ohio, USA, *710*, 120.
[2] D. M. Powers, *arXiv preprint arXiv:2010.16061* **2020**.
[3] K. M. He; G. Gkioxari; P. Dollar; R. Girshick, *IEEE I Conf Comp Vis* **2017**, 2980.
[4] N. Miljkovic; R. Enright; E. N. Wang, *ACS Nano* **2012,** *6*, 1776.
[5] S. Sett; P. Sokalski; K. Boyina; L. N. Li; K. F. Rabbi; H. Auby; T. Foulkes; A. Mahvi; G. Barac; L. W. Bolton; N. Miljkovic, *Nano Lett* **2019,** *19*, 5287.
[6] Q. L. Zhang; M. He; J. Chen; J. J. Wang; Y. L. Song; L. Jiang, *Chem Commun* **2013,** *49*, 4516.
[7] J. B. Boreyko; B. R. Srijanto; T. D. Nguyen; C. Vega; M. Fuentes-Cabrera; C. P. Collier, *Langmuir* **2013,** *29*, 9516.
[8] S. M. Kelleher; O. Habimana; J. Lawler; B. O'Rilly; S. Daniels; E. Casey; A. Cowley, *ACS Appl Mater Inter* **2016,** *8*, 14966.
[9] N. Miljkovic; R. Enright; Y. Nam; K. Lopez; N. Dou; J. Sack; E. N. Wang, *Nano Lett* **2013,** *13*, 179.
[10] X. Yan; L. Zhang; S. Sett; L. Feng; C. Zhao; Z. Huang; H. Vahabi; A. K. Kota; F. Chen; N. Miljkovic, *ACS Nano* **2019,** *13*, 1309.
[11] R. Enright; N. Miljkovic; J. L. Alvarado; K. Kim; J. W. Rose, *Nanosc Microsc Therm* **2014,** *18*, 223.
